# Sometimes Less Is Not Enough: A Commentary on Greiff et al. (2015)

**DOI:** 10.3390/jintelligence5010004

**Published:** 2017-01-05

**Authors:** André Kretzschmar

**Affiliations:** 1 Hector Research Institute of Education Sciences and Psychology, Eberhard Karls Universität Tübingen, 72072 Tübingen, Germany; kretzsch.andre@gmail.com; Tel.: +49-0-7071/29-76529; 2 School of Psychology, University of Western Australia, Crawley WA 6009, Australia

**Keywords:** complex problem solving, assessment, multiple complex systems, validity

## Abstract

In this commentary, I discuss some critical issues in the study by Greiff, S.; Stadler, M.; Sonnleitner, P.; Wolff, C.; Martin, R., “Sometimes less is more: Comparing the validity of complex problem solving measures”, *Intelligence* **2015**, *50*, 100–113. I conclude that—counter to the claims made in the original study—the specific study design was not suitable for deriving conclusions about the validity of different complex problem-solving (CPS) measurement approaches. Furthermore, a more elaborate consideration of previous CPS research was found to challenge Greiff et al.’s conclusions even further. Therefore, I argue that researchers should be aware of the differences between several kinds of CPS assessment tools and conceptualizations when the validity of CPS assessment tools is examined in future research.

## 1. Introduction

Complex problem-solving (CPS) skills involve human interaction with problems that are characterized by features such as intransparency, dynamics, and complexity [1]. As our world is becoming increasingly complex and dynamic, CPS is viewed as an important 21st century skill, and research on CPS tends to attract a great deal of interest [2,3,4]. It is noteworthy that research on CPS has always been greatly influenced by the psychometric quality of the assessment tools that are used (for an overview, see [5], and also the recent discussion of [6,7,8,9,10]). Greiff, Stadler, Sonnleitner, Wolff, and Martin’s study [11] 1 on the validity of different CPS assessment tools therefore offers an important contribution to the assessment of cognitive abilities and, in particular, to the field of research on CPS.

More specifically, Greiff et al. [11] compared two approaches that are used in the assessment of CPS: one building on multiple complex systems (MCS) and the second based on classical measures of CPS via more complex computer simulations. The authors presented a fair selection of assessment tools that differed in many features, such as complexity (see [6]). The general finding of Greiff et al.’s study was that CPS assessment tools that are based on the MCS approach (i.e., MicroDYN [13], MicroFIN [14], Genetics Lab [15]) should be considered more valid than classical measures of CPS (i.e., Tailorshop [16]). As classical microworlds have dominated the CPS research field for decades, and the MCS approach was developed only quite recently, Greiff et al.’s conclusion about the validity of the different CPS measurement approaches might lead to a change in the standard assessment procedure that is applied in the CPS research field.

However, a closer examination suggests that Greiff et al.’s comparison of instruments might have been compromised by several difficulties, which will be highlighted in this commentary. These issues are related to (1) the Tailorshop assessment instrument and its application; (2) the MicroFIN assessment instrument and its application; (3) the statistical analyses; and (4) the interpretation of the results and their relations to previous research. Consequently, I will argue in this commentary that Greiff et al.’s conclusions should be considered critically and subjected to further research. In this sense, the aim of the present commentary is to offer information that will help provide a more elaborated perspective from which to evaluate Greiff et al.’s findings and conclusions.

## 2. Issues Related to the Tailorshop Assessment Instrument

For the last 20 years, assessments of CPS performance have usually involved a multistage procedure [17]. This means that participants first have to explore the system in order to acquire knowledge about it; then, the acquired knowledge is tested with a knowledge test; and, finally, the participants have to apply their knowledge to solve the problem. This procedure is common in MCS assessment tools (e.g., MicroDYN [13], MicroFIN [14], Genetics Lab [15]) and in classical CPS assessment tools (e.g., FSYS [18], Tailorshop [19], LEARN! [20], PowerPlant [21]). Exceptions can be found in previous CPS studies, for example, a reversal of the order of presentation of the knowledge test and knowledge application [22], exploration without a knowledge test [23], and passive instead of active exploration [24]. However, the processes of knowledge acquisition (i.e., exploration and knowledge assessment) and knowledge application (i.e., achieving goals; often called the control performance) reflect the main characteristics of CPS and are considered in MCS and classical CPS assessment approaches (e.g., [25,26,27]). It is noteworthy that the application of the Tailorshop instrument in Greiff et al.’s study did not include an exploration phase or a knowledge test.

### 2.1. Missing Exploration Phase

An exploration phase was applied only in the MCS assessment tools (i.e., MicroDYN, Genetics Lab, and MicroFIN) but not in the Tailorshop test. Therefore, participants were able to freely explore the CPS tasks in the MCS assessment tools but were not allowed to explore the Tailorshop simulation before being asked to increase the company’s value in the knowledge application phase.

It should be noted that omitting the exploration phase can have substantial implications for the cognitive demands and task difficulty involved in the Tailorshop assessment. Kluge [17] mentioned that the absence of an exploration phase leads to learning “under the gun” (p. 286) due to the paradoxical situation of not having the kind of knowledge that is needed to achieve the goals but simultaneously being required to achieve the goals. Without an exploration phase, participants have to simultaneously acquire information about how to reach the goals, integrate this knowledge into their behavior after each interaction, and achieve the goals in a limited number of steps. Thus, it can be concluded that substantially higher cognitive demands were placed on the participants in Greiff et al.’s Tailorshop assessment in comparison with the MCS tests. 2 Moreover, as the risk of making a mistake is always high in the early stages of problem solving (i.e., when the problem situation is unknown [28]), the approach of combining the knowledge acquisition and knowledge application phases in the Tailorshop assessment reduced the probability of success in solving the problem. For example, let us assume that a participant gets the first four steps of the problem-solving process wrong because no sufficient knowledge is available. The participant will then most likely use these four steps to gather knowledge about how to reach the goals. The participant will then probably use this knowledge to actually solve the problem, but the limited number of the eight remaining steps that are left available for achieving the goals might not be enough to compensate for the first four incorrect steps. Thus, this approach, as applied in Greiff et al.’s study, most likely increased the overall difficulty and may have decreased the reliability as well.

Furthermore, it is important to emphasize that previous research has repeatedly demonstrated the importance of a separate exploration phase when assessing CPS performance (e.g., different exploration behavior between a non-exploration group and an exploration group, see [29]; for an overview of the different impacts on acquired knowledge and the control performance, see [17]).

### 2.2. Missing Knowledge Test

The second critical issue concerning the application of the Tailorshop assessment is also related to the knowledge acquisition phase. As mentioned above, both processes (i.e., knowledge acquisition and knowledge application) were assessed in the MCS assessment tools, but the Tailorshop assessment was limited to an investigation of only knowledge application (i.e., no test of knowledge was administered in the Tailorshop assessment). According to Greiff et al., this approach was justified because “attempts to score the knowledge acquisition phase of the Tailorshop were found to be unreliable” [11] (p. 106).

This explanation is somewhat surprising in light of previous research. In fact, a content-valid knowledge test for Tailorshop with sufficient test-retest reliability (e.g., *r* = 0.70 [30]; *r* = 0.67 [31]) and internal consistency (Cronbach’s α > 0.71 [27]) has been used in previous studies. Moreover, the studies cited by Greiff et al. utilizing Tailorshop as an assessment instrument, first, did not report the reliability of the knowledge acquisition assessment and, second, never recommended that only the knowledge application phase of Tailorshop be used [19,32]. 3

The (non-)application of a knowledge test has an impact on the overall CPS performance because of reactivity to the knowledge test. Reactivity in this context means that by taking a knowledge test, participants become informed about features of the problem situation and may be stimulated to think about the problem situation and its solution. Consequently, the administration of a knowledge test before the knowledge application phase leads to more CPS task knowledge that can subsequently be used in the knowledge application phase (see the differentiated findings of [31,34]). Blech and Funke even interpreted a knowledge test as not merely an assessment tool but rather as an integrative part of CPS assessments [34]. Therefore, as there was no knowledge test in the Tailorshop assessment, but there was one in each MCS assessment tool, it was more difficult for the participants to work with Tailorshop compared with the MCS assessment tools. Thus, Greiff et al.’s approach of excluding the knowledge test may have led to increases in the difficulty of their Tailorshop assessment.

In conclusion, there were substantial differences between the two types of CPS assessment tools that were employed in Greiff et al.’s study. It is important to note that these differences were not based on genuinely different CPS measurement approaches but on the design applied in Greiff et al.’s study. It is uncertain whether the findings would remain the same if Tailorshop had been presented in a manner that was comparable to the MCS tests as well as to many previous Tailorshop studies. Hence, Greiff et al.’s findings on Tailorshop cannot be generalized to the Tailorshop assessment as applied in other studies or even to the classical CPS approach in general.

## 3. Issues Related to the MicroFIN Assessment Instrument

In addition, there are also issues related to the MCS assessment and, more specifically, to the application of the MicroFIN test. In general, the rationale behind the development of MicroFIN was the need to develop a test that could cover more heterogeneous tasks in comparison with established MCS-based instruments (e.g., MicroDYN or Genetics Lab) [14]. In fact, MicroDYN and Genetics Lab are characterized by a high degree of similarity: both are based on linear structural equations with the same advantages and limitations [14], employ the same optimal strategy for solving the tasks (VOTAT [29]), implement very comparable task demands, and have similar user interfaces. Consequently, it was important for Greiff et al. to include the more different MicroFIN test in their study to have a valid representation of the MCS approach.

However, in order to ensure that MicroFIN was presented as a reliable and heterogeneous assessment instrument, it would have been necessary to include several MicroFIN tasks. It is therefore uncertain whether a MicroFIN test with only two tasks as applied in the Greiff et al. study could adequately reflect the MCS principles (e.g., increased reliability on the basis of multiple tasks [13]) 4 and the MicroFIN approach (e.g., heterogeneity of the different tasks [14]). Although the small number of MicroFIN tasks was acknowledged by Greiff et al., the consequences for the study results were not sufficiently realized. On the one hand, increasing the number of MicroFIN tasks may have increased the reliability. On the other hand, as MicroFIN was developed to reduce the gap between the MCS-based assessment tools and classical CPS tests such as Tailorshop (see [7,10]), it is not unlikely that Greiff et al. would have found a substantially higher correlation between a more appropriate version of MicroFIN and the Tailorshop test, and this would have contradicted the claim that MCS tests share more common variance with each other than they do with classical CPS assessment tools.

In conclusion, including a comprehensive assessment of CPS via MicroFIN might have resulted in different findings and conclusions when considering the relation between the assessment tools of the MCS approach and Tailorshop.

## 4. Issues Related to the Analyses

Irrespective of the concerns outlined above, issues with the statistical analyses should be considered.

### 4.1. Research Question 1

For Research Question 1 (i.e., whether correlations between the different MCS tests were higher than those between the MCS tests and the classical instrument, Tailorshop), the statistical approach that was chosen was a comparison between models that was based on $\chi^{2}$ difference tests. More specifically, for the case in which reasoning was partialled out and for the case in which it was not partialled out, two models were compared: a restricted model with equal correlations between all variables (MCS tests and Tailorshop) and a less restricted (baseline) model with two values for the correlations, one value for the correlations between the MCS tests and another value for the correlations of the MCS tests and the Tailorshop variable. The model comparison was based on a $\chi^{2}$ difference test and was used to test whether the correlations between the MCS tests were higher than the correlations of these tests with the Tailorshop variable. The problem with this model comparison strategy is that for the $\chi^{2}$ difference test to be valid, at least the less restricted model needs to be a model that is considered to have good fit (e.g., [38]). Unfortunately, for the case in which the influence of reasoning is partialled out (Models 4 [11]), the less restricted (baseline) model did not fit well enough according to the goodness-of-fit tests reported by Greiff et al. [11] and common cut-off values [39].

Furthermore, when I followed Steiger’s [40] approach to test for differences between correlations (based on Table 2 in [11]), I found that when reasoning was not controlled for (Models 3 [11]), the assumption of equal correlations between the MCS tests held only for *r*_MicroFIN.GeneticsLab_ as compared with *r*_MicroFIN.MicroDYN_ (*z* = 0.67, *p* = 0.50). I found that *r*_MicroFIN.GeneticsLab_ as compared with *r*_GeneticsLab.MicroDYN_ (*z* = 2.88, *p* = 0.004) and *r*_MicroFIN.MicroDYN_ as compared with *r*_GeneticsLab.MicroDYN_ (*z* = 3.56, *p* < 0.001) differed significantly, thus contradicting the assumption of equal correlations between MCS tests. Furthermore, statistically significant differences were found for all correlations between the Tailorshop and the MCS tests (*z* > 2.1, *p* < 0.037). For the case in which the effect of reasoning was partialled out (Models 4 [11]), a similar pattern was found. Therefore, the model comparison as reported by Greiff et al. [11] is not a strong basis for the conclusion that the correlations between the MCS tests are higher than between the MCS tests and the Tailorshop variable.

### 4.2. Research Question 2

With regard to Research Question 2 (i.e., whether MCS tests show incremental validity beyond Tailorshop), Greiff et al. performed two different analyses: (1) they compared correlations between school grades and MCS tests with correlations between school grades and Tailorshop; and (2) they computed regression analyses for each MCS test in order to explain variance in school grades beyond Tailorshop.

Regarding the first approach (i.e., comparing correlations), the impact of intelligence—as an important predictor of CPS [41] and academic achievement [42]—should be controlled for (see e.g., [35,43]). In doing so, it is important to highlight Greiff et al.’s finding that MicroFIN and Genetics Lab were significantly and weakly correlated with school grades in the natural sciences (*r* ≤ 0.22). However, MicroDYN (as a prominent representative of the MCS approach) and Tailorshop had nonsignificant and negligible (*r* ≤ 0.13) correlations with school grades in the natural sciences when fluid intelligence was controlled for (see the partial correlations in Table 2 [11]). Furthermore, additional re-analyses revealed that comparing the average partial correlation between the MCS tests and natural science grades (*r* = 0.18) with the partial correlation between Tailorshop and natural science grades (*r* = 0.12) led to a nonsignificant difference (*z* = 0.8, *p* = 0.420). Therefore, there does not seem to be a clear pattern in which one is more predictive than the other.

On the basis of the second approach (i.e., the regression analyses), Greiff et al. argued that Tailorshop did not explain unique variance in school grades when MicroDYN and Genetics Lab were considered (see regression Models 5b to 5d [11]). Consequently, they concluded that the MCS tests have a higher incremental validity than the classical microworlds.

Although Greiff et al. [11] correctly mentioned that more sophisticated analyses (e.g., [44], another approach is the bifactor model [45]) are necessary for examining unique variance in the different CPS measures, the authors nevertheless interpreted their findings in terms of unique variance. It should be emphasized that based on the correlated first-order factor model, as applied in Greiff et al., no conclusions about the unique variances of the latent factors are warranted. In fact, each latent factor represents a conglomerate of common variance between the CPS measures (*g*-factor variance) and the specific variance of each CPS measure (unique variance; for the impact of the *g*-factor in a correlated factor model, see e.g., [46]). 5 Therefore, the analyses presented in Greiff et al. [11] were not sufficient for interpreting the unique variance of the different CPS measures (see [5] for a discussion about different measurement models in CPS research). Interpretations such as “Tailorshop no longer explained any unique variance” [11] (p. 111) are not justified and thus cannot be used as evidence against the validity of Tailorshop.

In conclusion, the results and interpretations as reported by Greiff et al. (i.e., that MCS tests have higher incremental validity and that they assess a broader CPS skill than classical CPS tests do) are not as clear as suggested. 6

## 5. Issues Related to the Interpretation of the Results and Their Relations to Previous Work

With regard to the construct validity of CPS (Research Question 1), Greiff et al. emphasized that usually small or nonsignificant correlations between classical CPS measures have been found [48,49,50], whereas MCS measures have been found to be substantially correlated with each other (as in the study by Greiff et al.), and especially when intelligence measures were controlled for. Greiff et al. took these findings as evidence against the validity of classical CPS measures and as evidence for the validity of the MCS tests.

It is important to note that two very different operationalizations of intelligence were used in the cited studies that featured classical CPS measures [48,49,50] 7 and Greiff et al.’s study [11]. Whereas the former used a comprehensive and construct-representative operationalization of intelligence (e.g., BIS test with different task contents and different facets of intelligence [51]; for a description in English, see [52]), the latter used a non-construct-representative operationalization (i.e., figural reasoning tasks from the IST 2000 R test [53]). It is obvious and it was empirically demonstrated (see [5]) that a comprehensive operationalization of intelligence can explain much more variance in CPS performance than a very specific operationalization. Therefore, it should be taken into consideration that it is possible that lower common CPS variance was found in the cited studies because a construct-representative operationalization of intelligence was applied in comparison with Greiff et al.’s study, in which only figural reasoning as a non-construct-representative operationalization was used. Consequently, the differences between the correlational patterns in the cited studies featuring classical CPS measures [48,49,50] and the results of Greiff et al.’s study [11] may also have been substantially influenced by different operationalizations of intelligence. Thus, a direct comparison of the results is not as straightforward as suggested. In fact, it is unclear whether the convergent correlations between the MCS tests would have been superior to the convergent correlations between classical microworlds if a construct-representative operationalization of intelligence had been used in Greiff et al.’s study.

Furthermore, there is an additional crucial difference between the study featuring classical CPS tests and Greiff et al.’s study. Süß’s study [48] was cited several times to illustrate that three different classical CPS measures showed no significant correlation after fluid intelligence was controlled for. It is noteworthy that in Süß’s study [48], when assessing CPS performance with each of the classical CPS measures, the author controlled not only for fluid intelligence but also for the part of the CPS performance that was due to knowledge acquisition. 8 To compare Süß’s findings [48] with Greiff et al.’s findings [11], only the variance that was unique to knowledge application after partialling out the variance that was due to knowledge acquisition in each MCS test should be considered, but this approach was not applied in any of the recent CPS studies featuring the MCS assessment tools. Given the high correlations between knowledge acquisition and knowledge application in the MCS tests (*r* = 0.83–0.93; see [12]), it is reasonable to question whether the findings would be any different from Süß’s findings [48].

In conclusion, unfortunately, both issues (i.e., the differences in the operationalization of intelligence and partialling out knowledge acquisition performance) were not considered in Greiff et al.’s discussion of their findings. In fact, the approaches taken in Greiff et al.’s study [11] and the aforementioned previous studies that applied classical CPS measures [48,49,50] are conceptually very different and, thus, hardly comparable. Conclusions about the construct validity of different CPS assessment approaches cannot easily be derived from this comparison.

## 6. General Conclusions

Since the development of the MCS approach and the corresponding new CPS measurements (i.e., MicroDYN [13], MicroFIN [14], and Genetics Lab [15]), research on CPS has attracted considerable interest (e.g., CPS tasks in the PISA 2012 study [4]). At the same time, a primarily theoretical discussion about the different measurement approaches has ensued (see [6,7,8,9,10]). Greiff et al.’s study [11] was the first to empirically examine relations between the new and the classical CPS measurement approaches. Thus, a study such as theirs is crucial for gaining a deeper understanding of the relations between different CPS assessment tools and their impact on the CPS research field in general.

However, comparing assessment instruments from different approaches requires the careful consideration of a range of factors involving the selection and application of specific instruments, the adequate analyses of empirical results, and the integration of the findings into the broader research landscape. Greiff et al.’s study [11] provided a first comparison, but generalizations with regard to other (and more adequate) versions of Tailorshop, the MicroFIN test, the MCS approach, or the classical CPS measurements as a whole are not yet warranted. The authors’ arguments that “MCS tests would provide a more valid measurement of CPS than classical measures” and “MCS tests seem to assess a broader CPS skill” [11] (p. 111) seem premature. My hope is that the issues raised in this commentary will be considered when the validity of different CPS tests is evaluated and, especially, when future studies that apply several CPS measurement approaches are conducted.

## Footnotes

1 Please note that Greiff et al. [11] reported extended analyses of a previous study [12]. Therefore, information from both studies was considered when necessary.

2 The common procedure applied in the MCS assessment tools allowed participants to freely explore each task to acquire knowledge without any goal except to explore the task and to use their knowledge to achieve several goals in the subsequent phase of the assessment. Thus, the cognitive demands were split and successively requested in the MCS tasks.

3 More specifically, the Tailorshop knowledge test was administered in one of the cited studies but was not included in the analyses (see [19]). The reason was the scope of the article and not the insufficient reliability of the knowledge test [33].

4 An examination of previous literature revealed that five to six tasks are the very minimum numbers of tasks that are usually employed in the MCS approach, independent of the specific operationalization (see e.g., [13,35] for MicroDYN; [5,14] for MicroFIN; [36,37] for Genetics Lab). Furthermore, the low reliability of the applied MicroFIN test (see Table 2 [12]) as well as issues with the measurement model (see [12]) can be taken as evidence against the adequacy of the MicroFIN version that was applied.

5 Greiff et al.’s finding that neither Tailorshop nor MicroFIN were significant predictors of school grades in a simultaneous regression (see Model 5c [11]) emphasized the impact of *g*-factor variance in a correlated factor model.

6 Please note also that Greiff et al. [11] cited Süß [27] several times with regard to the relation between Tailorshop performance and school grades. However, no such information was provided by Süß [27]. In fact, to date, there is little information in the literature on whether and to what extent a participant’s Tailorshop performance can be used to explain variance in school grades. However, there is evidence that Tailorshop performance can be used to incrementally explain variance in supervisory ratings beyond reasoning [32,47], a finding that does not yet appear to have been replicated with MCS assessment tools.

7 Please note that References [48,49,50] are partly based on the same study. Therefore, information from all references was considered when necessary.

8 The rationale behind this approach was the need for a different conceptualization of CPS. Broadly speaking, knowledge acquisition was considered part of (crystallized) intelligence and, thus, was not viewed as a specific type of CPS performance (see [27]).
